# The Effects of Processing Conditions and Pressure on Composite Polymer Electrolyte Performance

**DOI:** 10.3390/gels11110890

**Published:** 2025-11-05

**Authors:** Samantha P. Macchi, Lillian N. Elam, Josefine D. McBrayer, Noah B. Schorr

**Affiliations:** Power Sources R&D, Sandia National Laboratories, Albuquerque, NM 87185, USA; spmacch@sandia.gov (S.P.M.); lnelam@sandia.gov (L.N.E.); jdmcbra@sandia.gov (J.D.M.)

**Keywords:** gel polymer electrolyte, PVDFHFP, pressure, conductivity

## Abstract

Solid polymer and composite polymer electrolytes have been investigated as a replacement for liquid electrolytes in lithium batteries and have shown promising conductivities at room temperature. However, the literature reports often do not fully investigate the effects of residual solvent or testing apparatus conditions, leading to discrepancies in reported performance and possible conflation of conductivity values. Using combinations of poly (vinylidene fluoride-co-hexafluoropropylene), succinonitrile, and lithium lanthanum zirconium tantalum oxide, this work aims to develop an understanding of how polymer electrolyte composition, including solvent retention, affects conductivity. A custom-designed controllable pressure test fixture was utilized to determine ionic conductivity of the composite polymer electrolyte films at a wide range of pressures (1.5–18.7 psi) and temperatures 10–90 °C. Applied pressure during testing greatly influences apparent conductivities, with optimal composite film conductivity values ranging from 1.2 × 10^−5^ to 4.1 × 10^−5^ S cm^−1^ at room temperature. At elevated temperatures, the composite films achieved greater than 1 mS cm^−1^. The ideal pressure was found to be dependent on the polymer electrolyte additives. Symmetric cell testing showed over 99% Coulombic efficiency for over one hundred cycles at 0.1 mA cm^−2^. The results of this work highlight the importance of careful characterization of electrolyte films and controlled test fixture pressure when developing polymer electrolytes.

## 1. Introduction

Solid polymer electrolytes (SPEs) have been regarded as a promising replacement for the liquid electrolyte and separator in secondary lithium batteries [1,2]. However, the use of this terminology across the literature can be misleading, as many of these SPEs are not exclusively “solid”. Many reported SPEs require the addition of a small volume of liquid electrolyte in order to yield conductivities that are commercially relevant [3,4]. Additionally, common drying protocols are typically not sufficient to remove all of the casting solvent, leading to residual liquid content in the films. Recent reports have uncovered that for many SPEs, this residual solvent within the polymer matrix dominates the lithium conduction mechanism, especially for vinylidene fluoride-based SPEs [5]. The effectiveness of an SPE depends on many factors, such as crystallinity, mobility, mechanical strength, and lithium-cation-coordinating ability of the polymer(s). However, if residual solvent dominates lithium ion movement, deconvoluting the effect of changes in other properties can prove difficult.

Poly (vinylidene fluoride-co-hexafluoropropylene) (PVDF-HFP) has gained traction as a promising SPE matrix due to ideal properties such as low glass transition temperature and electrochemical stability [6]. However, PVDF-HFP has received criticism in recent years due to the lack of Li^+^ coordination ability of the fluorinated polymer [7]. Because vinylidene-based polymers are unable to solvate lithium cations directly, many literature reports of “SPEs” have been developed by combining PVDF/PVDF-HFP and plasticizing solvents. However, high boiling point solvents such as N,N-dimethylformamide (DMF), dimethylsulfoxide, N-methylpyrrolidine (NMP), etc., are difficult to fully remove from the polymer matrix, even under vacuum at relatively high temperatures. Additionally, many reports either do not express specifics of drying conditions or do not attempt to fully dry the films (dried without vacuum or dried far below the solvent boiling point). Despite the well-defined correlation between residual solvent and conductivity of SPEs, many reports lack transparency in quantifying solvent retention or its influence on the high observed conductivity [8,9,10]. There is surprisingly little discussion regarding the effect of these solvents in the “solid” polymer electrolyte literature, though recently several groups have begun to investigate this disparity [7,11]. For polymer electrolytes that contain significant residual solvent, the polymer acts primarily as a passive vessel and thus are more accurately defined as gel polymer electrolytes (GPEs).

Besides residual solvent, we also discovered a lack of information in the literature regarding conductivity testing fixtures in the field of SPE/GPEs. Typically, coin cells or T-cells are used to sandwich the SPE between blocking electrodes to evaluate the electrochemical impedance spectroscopy (EIS) of SPEs. However, specific pressure data is often neglected. This detail is relevant to these measurements as compression can affect ion pathways, solvent distribution, SPE/GPE thickness, and contact of the SPE. To our knowledge, there is no study that investigates variable pressure and its effect on observed polymer electrolyte conductivity.

Additionally, composite solid polymer electrolytes (CSPEs) with the addition of inorganic fillers have been extensively studied with promising results. The addition of inorganic fillers (conductive or non-conductive) has been reported to achieve the best qualities of SPEs and inorganic solid electrolytes while avoiding the disadvantages of either [9,12]. Namely, the introduction of filler introduces alternative fast ion transport pathways while the polymer matrix allows for mechanically flexible and robust films [13]. Inorganic fillers have also been shown to aid in improving the interfacial stability of GPEs by suppressing side reactions of residual solvent [14]. Reports of composite polymer electrolytes indicate the positive effects of both plasticizers and inorganic fillers [15]. However, deconvoluting the effect of additives is difficult when solvent interactions must also be considered. Despite perceived advances in SPE and CSPE performance, there are many factors that have resulted in films of similar composition with exhibited differences in conductivity (i.e., optimal percentages of additives, residual solvent, etc.). Among them, SPE preparation conditions play a key role [7].

To better understand the combined effect of succinonitrile (SN) plasticizer, Li_6.4_La_3_Zr_1.4_Ta_0.6_O_12_ (LLZTO) filler, solvent retention, and applied pressure on the performance of GPE films derived from PVDF-HFP, we synthesized and characterized several iterations of polymer films, as well as used variable pressure techniques to study GPE conductivity. We found that the relationship between conductivity and pressure is non-linear for GPEs, and the relationship is highly dependent on the additives in the film. Because of this, one GPE exhibited the greatest conductivity at low pressure, while another prevailed at greater pressure. Additionally, the overall conductivity results corroborate the retained solvent-dominant ion pathways in GPEs prepared with a high-boiling-point solvent. From these results, we conclude that variation in reported conductivity values of polymer electrolytes is dependent on slight variations in the heat treatment, preparation, and the film pressure during testing conditions.

## 2. Results and Discussion

**Figure 1:**
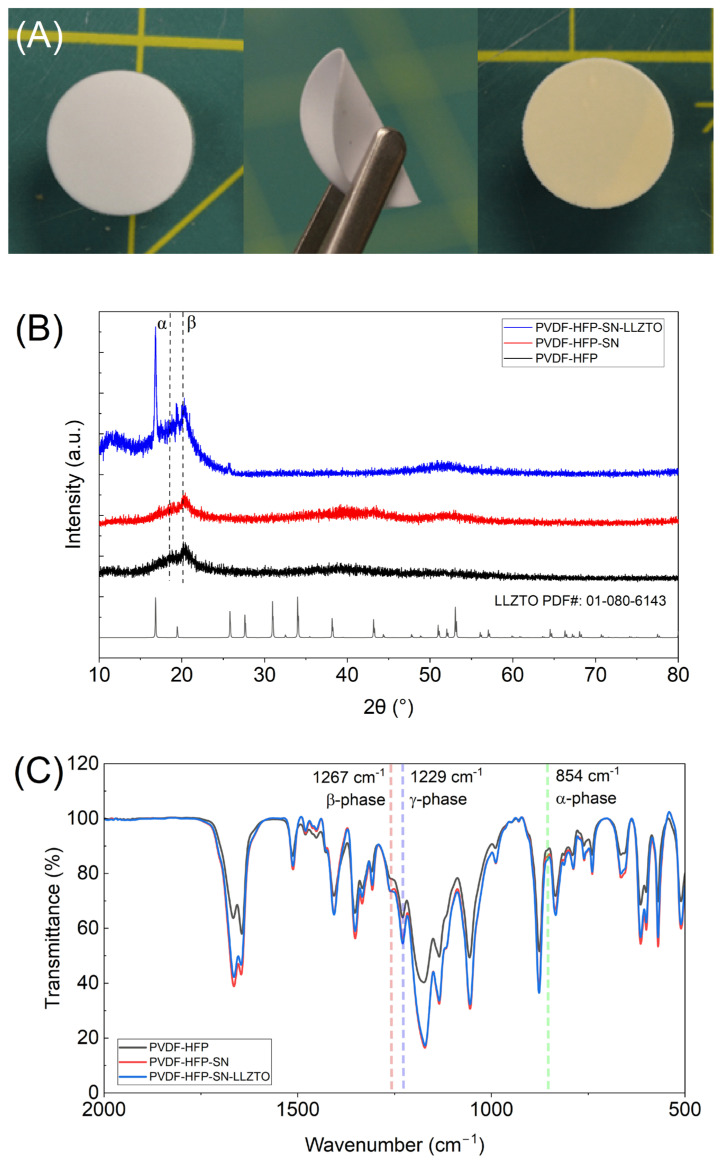
(**A**) Digital camera images of GPEs from left to right: PVDF-HFP, PVDF-HFP-SN, and PVDF-HFP-SN-LLZTO; (**B**) XRD spectra of the films; and (**C**) FTIR of GPEs.

Three films were developed utilizing PVDF-HFP as a polymer matrix and LiTFSI as a Li^+^ source. One included no additives (PVDF-HFP), one employed SN plasticizer (PVDF-HFP-SN), and one included both SN and LLZTO (PVDF-HFP-SN-LLZTO). All three films were opaque (Figure 1A) and possessed thicknesses of 299 ± 41 microns (Table 1). Solvent retention was calculated based on casted mass and final GPE mass (Table 1). Plasticization of PVDF-HFP by SN is likely responsible for the increase in solvent retention (>10%) due to enhanced solvent interactions [16]. Both GPEs with plasticizer exhibited NMP retention greater than 20%, even with vacuum drying at 70 °C, indicating the strong interactions between the solvent and SN. Additionally, all films were flexible and easily cut into any shape (Figure 1A). X-Ray Diffraction (XRD) and Fourier Transform Infrared Spectroscopy (FTIR) were used to characterize the phase and bonding environment of PDVF-HFP matrix in GPEs. Generally, all GPEs showed mostly amorphous character with broad peaks centered around 18.7° and 20.2°, corresponding to α and β-phases (Figure 1B) [17]. The observed deviation from pristine PVDF-HFP is due to residual NMP, which modifies the polymer structure [18]. Retention of solvent within the polymer matrix has been separately confirmed by FTIR and TGA and is discussed later. The XRD spectra of PVDF-HFP-SN-LLZTO showed several additional peaks, due to the incorporation of crystalline phases of LLZTO in the polymer matrix. Additionally, the PVDF peak intensity decreased upon addition of SN into the polymer matrix, indicating lowered crystallinity. The FTIR spectra of GPEs were very similar to one another (Figure 1C), showing typical peaks from PVDF-HFP and LiTFSI salt (Table 2). However, some differences could be seen. GPEs containing plasticizing SN show a slight shift to lower wavenumber for the peak at ~1173 cm^−1^ corresponding to –CF_2_– bonding of PVDF-HFP (Figure S1). This is due to the amorphization of PVDF-HFP, leading to lower energy vibration mode. Tertiary amines were correlated to peaks ~1676 cm^−1^ and were due to the presence of residual NMP in the GPEs [19]. The peak intensity of PVDF-HFP is much less than that with SN, indicating that inclusion of the plasticizing agent resulted in greater solvent retention. All three GPEs exhibited a similar strong peak at 1229 and 1267 cm^−1^, attributed to the β- and γ-phase [20,21]. The presence of α-crystalline character (~854 cm^−1^) [22] was also present in all three samples, but at low intensity compared to other phases. Composite XRD and FTIR results indicate that in the three films, PVDF-HFP exists mostly in an amorphous form.

**Figure 2:**
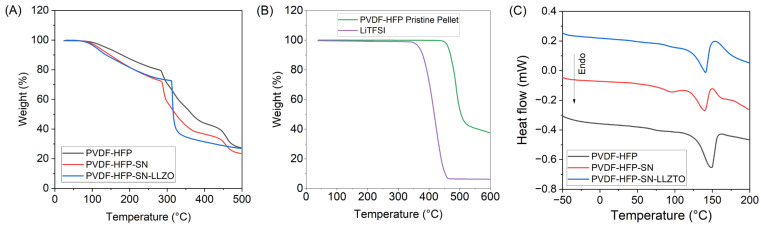
(**A**) TGA of GPEs, (**B**) TGA of raw materials, and (**C**) DSC curves of GPEs at a 10 °C min^−1^ ramp rate.

Thermogravimetric analysis (TGA) and differential scanning calorimetry (DSC) were employed to investigate the thermal properties of GPEs. The obtained TGA curves illustrated the thermal degradation behavior of the GPEs (Figure 2A). All three films exhibited two significant mass loss events. This is contrary to either PVDF-HFP pristine polymer or LiTFSI alone, which show single-step decompositions at ~470 °C and 384 °C, respectively (Figure 2B). The decrease in onset temperature and divergence to multi-step decomposition of films compared to pristine components has been previously observed [23]. All three films exhibited two significant mass loss events. Initial weight losses were primarily due to the loss of solvent bound to the polymer matrix. Estimating solvent retention from this early-onset mass loss is common; however, for complex mixtures, these values can be misleading due to the convolution of other degradation events. Solvent retentions determined from mass percent remaining at the end of the first decomposition event are slightly larger than the calculated values from film preparation for all GPEs. This is more exaggerated for PVDF-HFP GPE due to the earlier onset (<300 °C) of decomposition of PVDF-HFP in the absence of additives [24]. Films with no additives and PVDF-HFP-SN exhibit lower onset temperatures compared to those with LLZTO incorporation, indicating that LLZTO can strengthen solvent interactions in the GPE. Mass losses past ~300 °C were attributed to preliminary decomposition of PVDF-HFP and lithium bis (trifluoromethane sulfonyl)imide (LiTFSI) salt [25]. To understand changes in enthalpy, melting, and crystallinity upon the addition of additives, DSC was performed (Figure 2B). Enthalpy and percent crystallinity, X_c_, are both greatest in the case of GPE with no additives. Additionally, these parameters are lowest for the PVDF-HFP-SN. This result indicates that, regardless of other additives, SN serves to increase the amorphous character of the PVDF-HFP. Introduction of LLZTO slightly increases film crystallinity, but not to the degree of GPE with no plasticizing agent. These findings support previous XRD and FTIR data regarding polymer crystallinity. The melting temperature of PVDF-HFP in GPEs followed a similar trend to polymer crystallinity. However, the melting temperature of SN was not altered substantially upon LLZTO incorporation, indicating little interaction between the two.

To determine polymer electrolyte conductivity, two stainless steel blocking electrodes sandwiched the GPE in either a coin cell, T-cell, or custom apparatus. These cell formats each differ in the degree and ease of quantifiability of the pressure applied to the GPE and come with their own set of challenges. The resulting pressure in coin cells is not universal because components (spacer number/thickness, type of spring) vary among manufacturers, and assembly order is dependent on the researcher. Additionally, the compressed thickness of the GPE film in the coin cell cannot be measured, complicating both the conductivity calculation and replicability. When using a T-cell, however, the pressure is applied by hand and is difficult to replicate consistently. Initially, EIS was recorded using both a Swagelok-type T-cell and a typical 2032-coin cell, and was then compared (Figure 1A). Variation in the Nyquist plot was observed between the two methods for PVDF-HFP GPE. The change in resistance between methods does not necessarily correlate to a change in the resultant conductivity, due to the likelihood that the compressed thickness was different between the two methods. Additionally, a non-Arrhenius behavior was observed using a T-cell at variable temperature (up to 60 °C, Figure S1). We hypothesized this could have been due to an issue with pressure exhibited on the GPE during testing.

**Figure 3:**
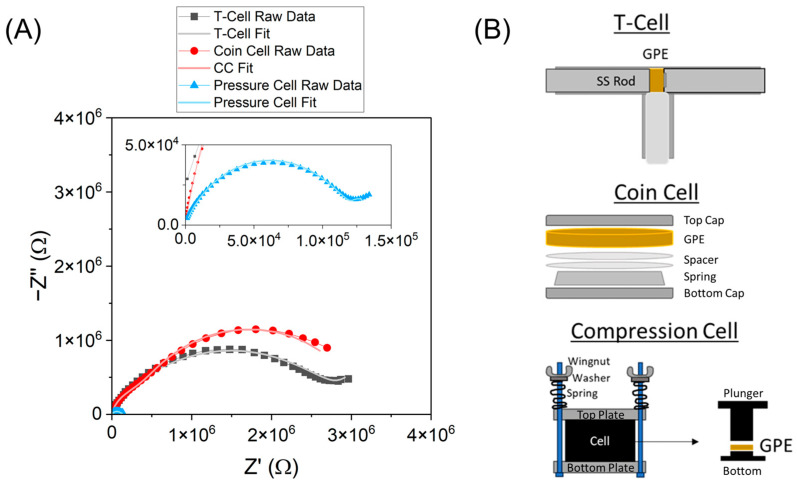
(**A**) Nyquist plots of various EIS test cell formats for PVDF-HFP GPEs and (**B**) schematic of the three apparatuses used for initial EIS testing.

Due to the aforementioned issues, we sought out an alternative test fixture, which would allow the applied pressure to be tunable and the compressed film thickness measurable. To understand the effect of cell pressure on film conductivity, GPEs were tested in an MSE polyether ether ketone (PEEK) compression cell, modified to be able to tune pressure via the measurement of spring displacement (Figure 3B). With this apparatus, both the applied pressure and resultant film thickness can be determined. Figure 3A shows that the resistance values are variable among the tested fixtures due to changes in pressure and film compression. If there is no change in conductivity upon compression, the resistance will vary inversely with thickness (Equation (1)). This is seen for the compression cell with high applied pressure (47.5 psi), which exhibits lower resistance than for the coin cell or T-cell. The effect of compression on resulting conductivity will be discussed in the following section.

**Figure 4:**
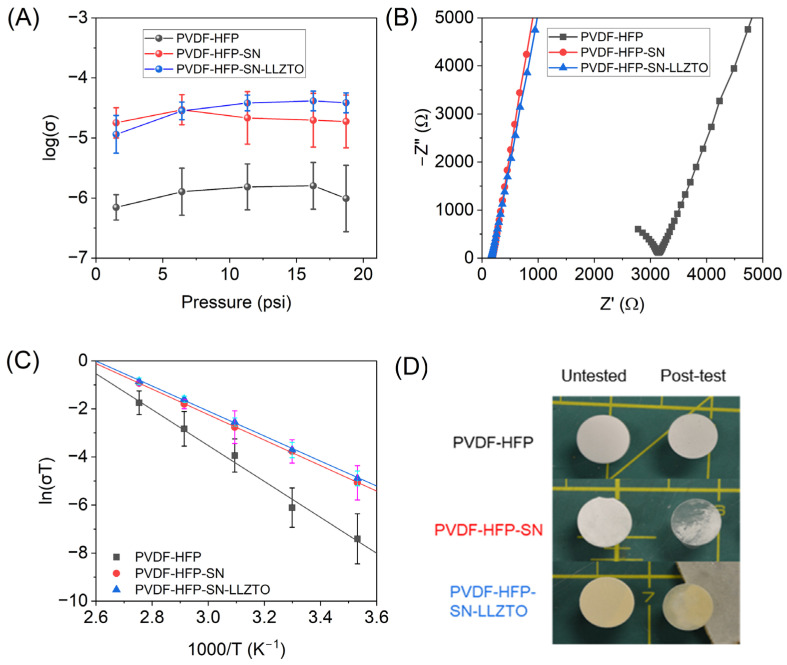
(**A**) Ionic conductivity of GPEs relative to cell pressure at room temperature and (**B**) Nyquist plot of GPEs at optimal pressure and at 30 °C, (**C**) Arrhenius plots of GPEs, (**D**) and digital camera photographs of GPEs before and after compression/temperature EIS studies.

We hypothesized that polymer structure could be altered depending on the degree of compression due to solvent interactions within the matrix. Conductivities were measured at five pressures from 1.5 to 18.7 psi in triplicate (Figure 4A). Standard error increased with pressure for PVDF-HFP and -SN films. We hypothesize that solvent displacement is inhomogeneous in the polymer films without inorganic filler, resulting in overall lower errors for PVDF-HFP-SN-LLZTO; likely, this film does not experience the same solvent displacement issues due to the presence of inorganic filler. For all films, increased pressure resulted in an increase in conductivity up to a maximum pressure, where conductivities either decreased or remained constant. The optimal pressure for the highest average conductivity was variable among GPEs, where films with no additives and PVDF-HFP-SN-LLZTO showed maximum conductivities at higher pressure (16.3 psi), but PVDF-HFP-SN exhibited a maximum at a lower pressure (6.4 psi). Figure S2 shows the dependence of compressed thickness on pressure. Compressed thickness for all films decreased with increasing pressure, but the dependence of pressure on the degree of compression varied among films at low to moderate pressures. At high pressures (>16.3 psi), the percent compression of all films converged around 50%. High solvent retention of PVDF-HFP-SN was seemingly the reason there were changes in polymer conformation/Li^+^ pathways under high pressures. The PVDF-HFP-SN and PVDF-HFP-SN-LLZTO films each had similar solvent retention, but the composite did not follow the same trend. This means that the inorganic LLZTO prevents the morphological/polymer reordering through interactions not present within the PVDF-HFP-SN. Figure 4A shows that the pressure applied can skew observed results; at very low pressure, PVDF-HFP-SN possesses the highest conductivity, while at the greatest pressure, the trend swaps with PVDF-HFP-SN-LLZTO. This highlights the importance of measuring cell pressure and film compression.

To study the temperature-dependent behavior of GPEs, films were compressed to optimal pressure (the highest average conductivity at room temperature) and remained unchanged during temperature testing (10–90 °C). The fixture was held at a temperature for a minimum of ten minutes prior to taking EIS measurements to allow for thermal equilibration. Plots for each GPE at various temperature points are given in Figure S3. The comparative plot at 30 °C shows increased resistance of GPE with no additives compared to the films with SN and SN-LLZTO (Figure 4B). Arrhenius plots of GPE ionic conductivities are shown in Figure 4C, and all GPEs studied exhibited linear Arrhenius behavior. Energy of activation, E_a_, was calculated from Arrhenius data via Equation (2) to further understand ion transport in GPEs. Plasticizer-containing GPEs possessed similar E_a_ values (0.46 and 0.45 eV, Table 3), both lower than PVDF-HFP films (0.66 eV). This correlates to a longer Li^+^ pathway of the sample with a lower amount of solvent retained in the structure [26]. The similar dependence of the -SN and -SN-LLZTO films with temperature and pressure indicates both films transport Li^+^ through the same mechanism.

Additionally, films with plasticizer (-SN and -SN-LLZTO films) showed distinct visual changes after undergoing simultaneous compression and heating (Figure 4D). Notably, there is a transparent character to each of the films post-EIS, which is inhomogeneously distributed. During testing, the GPEs are brought to temperatures above the melting point of the SN plasticizer (~55 °C). The color change and plasticizer properties support that the polymer film underwent restructuring at high temperatures under pressure, leading to an irreversible compression and change in solvent distribution during cooling. Interestingly, regardless of additives, the resistance after cooling is lowered (no change in applied force or volume, Figure S4). We hypothesize that isochoric heating of the films under pressure introduces permanent structural changes, which offer a more facile pathway for ion movement. This is more pronounced in the case of PVDF-HFP without additives and with the lowest solvent retention, indicating that molecular reorganization plays a more prominent role when there is less solvent present in the matrix.

From the cumulative conductivity data, we concluded that ion conduction in the studied GPEs is primarily through solvent interactions. From temperature-dependent conductivity data, there is no change in slope of the Arrhenius plot above the melting point of SN (~55 °C), indicating that a new liquid phase does not change the conductivity mechanism. This supports that retained solvent plays a dominating role in conductivity. However, the presence of SN, regardless of phase, alters the Li^+^ pathway. This is because SN itself can also coordinate with lithium ions, and increases the amount of retained solvent [27]. The SN plasticizer also influences the phase distribution and mobility of the PVDF-HFP [28]. Thus, ion pathways can be altered via additives to improve conductivity, but for GPEs containing solvent, the mechanism of ion conduction is not greatly affected by additives.

Compared to similar polymer electrolytes containing LLZO-based conductive filler, the PVDF-HFP-SN-LLZTO films cast in this study show a similar conductivity value at room temperature (Table 4). There is high variability in casting solvents and conductivity test cells in the literature reports. However, of those mentioned in Table 4, none of the studies include a solvent retention value or measured pressure. Most studies utilize a coin cell (size and closing force are variable), but the number and thickness of spacers used are not reported. Thus, the conductivity values reported for similar films are likely different due to differences in film preparation and how the pressure of the film was controlled. Without more specific details on the GPE preparation, there is no way to compare why film properties vary between reports.

**Figure 5:**
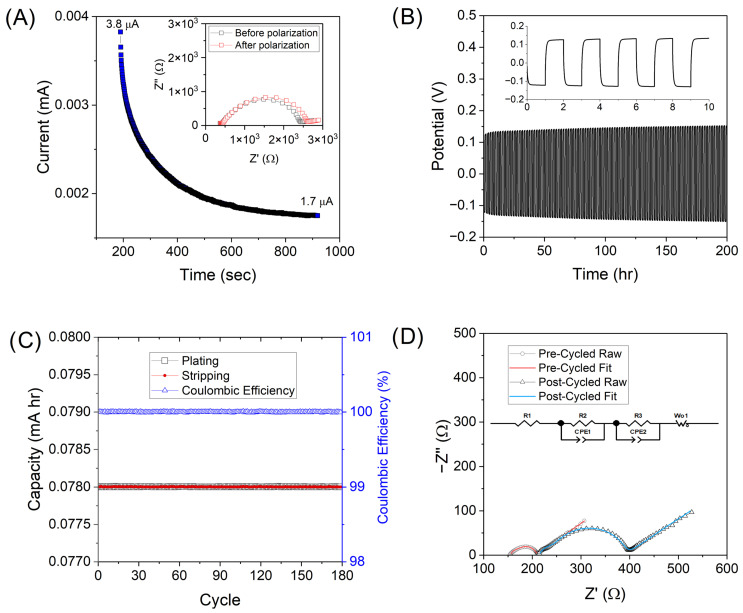
(**A**) Current–time profile of symmetric cell upon application of 10 mV DC voltage vs. OCV with inset showing change in impedance spectra before and after at room temperature, (**B**) symmetric cell cycling of PVDF-HFP-SN-LLZTO at a current density of 0.1 mA cm^−2^ and 50 °C with inset showing zoomed in view of the first five cycles, (**C**) plot of capacity and Coulombic efficiency for 180 cycles, and (**D**) Nyquist plots of symmetric cell before and after cycling, showing equivalent circuit model.

In addition to conductivity, the lithium transference number, t_Li+_, is a key parameter when assessing GPE performance. This value describes the ratio of lithium ion to total ion transference (Equation (3)). Experimental measurements utilized in the t_Li+_ calculation are shown in Figure 5A. A high t_Li+_ value can enable improved cell cycle life by reducing concentration polarization that leads to dendrite growth. Ceramic fillers like LLZTO have been shown to improve the t_Li+_ by hindering the movement of larger ions such as TFSI^−^ [33]. This is due to the Lewis acidic nature of the LLZTO surface and partial dehydrofluorination of PVDF-HFP [15]. However, the opposite trend was seen for GPEs prepared in this work (Table 3). Both GPEs with plasticizer showed a higher t_Li+_ than for PVDF-HFP alone (0.18). However, the greatest t_Li+_ (0.95) was obtained for PVDF-HFP-SN GPE, which is attributed to the formation of optimal anchored SN-Li^+^ associations, inhibiting the movement of the larger anions [34]. The plasticizer, SN, can polymerize upon interaction with La of LLZTO, causing encapsulation of LLZTO particles [35]. In the case of GPEs with LLZTO incorporated, a reduced amount of free SN in the polymer matrix, coupled with the SN-covered LLZTO unable to tether TFSI^-^ groups, both resulted in a decrease in t_Li+_. Other groups have seen a decrease in t_Li+_ when LLZTO is coated, particularly for thick coatings [36]. Additionally, LLZTO may increase polymer mobility, thus leading to an increase in ion pairing and subsequently lower charge carrier concentrations [37]. Increased mobility is supported by a shift in the FTIR corresponding to -CF_2_- and increased solvent retention. However, the total ion conductivity of both SN-containing GPEs remained similar, further confirming that solvent retention is the primary mechanism of cumulative ion conduction in the GPEs.

Due to its high conductivity, low E_a_, acceptable t_Li+_, and potential for improved interfacial stability, PVDF-HFP-SN-LLZTO GPE was selected to cycle in a symmetric Li|GPE|Li cell (Figure 5B). The interface stability between Li metal is a crucial parameter, as instability will lead to dendrite formation and eventual cell failure. The cell shows stable cycling over 180 cycles at 0.1 mA h cm^−2^ (Figure 5B,C) with coulombic efficiencies of ~100% throughout testing, highlighting the stability of the GPE even with considerable solvent retention. The stability of ceramic filler-containing polymer electrolytes is often attributed to the ceramic particle’s ability to act as a barrier, which prevents reactions at the interface of Li metal [38]. Also, the maximum overpotential increases from 0.126 to 0.153 V, resulting in an overall change of ~27 mV. In order to better understand this increase, EIS was performed. Impedance spectra were taken before and after cycling and fit using the equivalent circuit in Figure 5D, consisting of three resistors (R_x_), two constant phase elements (CPE_y_), and a Warburg element (W_O_) similar to previous work [39]. Resistance elements are attributed to bulk resistance of the GPE, solid-electrolyte-interphase (SEI) resistance, and charge-transfer resistance. CPE elements are related to SEI and double-layer capacitance, while the Warburg element describes Li^+^ ion diffusion. Diffusion properties remain similar before and after cycling, indicated by the angle of the Warburg tail in the Nyquist plot. Post-cycled EIS showed an increase in ohmic and charge transfer resistances, indicative of the formation of insulating products at the GPE|Li interface during cycling. This degradative process is well known and is due to the incompatibility of NMP with Li metal anodes. However, long-term stabilities comparable to traditional liquid LIBs, even in a full cell configuration, are unlikely. Methodologies to completely eliminate residual solvent while retaining ionic conductivity of composite polymer electrolytes should be investigated. Additionally, it is imperative to quantify the amount of residual solvent in SPEs/GPEs and the pressure applied by the apparatus used to collect impedance data, such that values can be comparable among labs.

## 3. Conclusions

Practical solid-state batteries cannot be realized until solid electrolytes with exceptional properties can be easily and reliably produced at scale. The body of literature surrounding “solid” polymer electrolytes prepared with high-boiling-point solvents has largely failed to quantify residual solvent and pressure effects on performance. Three GPEs were prepared with and without additives, and the combined effect of plasticizer/filler and retained NMP was evaluated. The GPE prepared with SN and LLZTO achieved room temperature conductivity of 4.14 × 10^−5^ S cm^−1^ and exhibited stable cycling in a Li symmetric cell over 180 cycles (15 days). While plasticization can improve performance by further amorphization of PVDF-HFP, solvent retention dominates the high conductivity of GPEs. Future work in this area should focus on methods to eliminate residual solvent while retaining the high ionic conductivity of solid polymer films. In contrast to many literature reports, LLZTO was not correlated to a considerable increase in ionic conductivity, and its influence on preparation conditions should be further studied. Finally, we found that the resultant conductivity is greatly influenced by applied pressure in the chosen test fixture. Thus, we recommend that reporting of pressure, temperature, and retained solvent parameters be mandatory to enable accurate comparisons of conductivity values for polymer electrolytes.

## 4. Materials and Methods

### 4.1. Film Preparation

PVDF-HFP (M_n_ = 455 k) was separately dissolved in NMP under magnetic stirring for 2 h at 60 °C at 15 wt% loading in solvent. Then, LiTFSI salt was added to each mixture at a weight ratio of 2:1 and further stirred to acquire a homogeneous solution. All solutions were prepared in an Argon-filled glove box (<1 ppm O_2_, <1 ppm H_2_O). These solutions were then cast into plastic molds and dried in a fume hood located within a moisture-free dry room (dew point < −55 °C) for 48 h at room temperature. After initial drying, films were removed from molds and vacuum dried at 70 °C and 215 mbar for 12 h. To develop composite GPEs, the ratio of polymer to salt remained fixed, and SN was added at a mass fraction of 10%. The solutions were magnetically stirred at 60 °C for an additional hour, and the casting process was repeated. Lastly, the ratios of polymer to salt to plasticizer were unmodified, and conductive filler, LLZTO (400–600 nm), was added at a mass fraction of 5%. The mixture was once again magnetically stirred at 60 °C for one hour and drop cast into the mold. The slurry masses were recorded before and after drying to determine the approximate solvent retention of the final films.

### 4.2. GPE Physical Characterization

Thermal decomposition data were collected using TGA (TA Instruments, Discovery 5500, New Castle, DE, USA). Thermograms were collected under an argon flow rate of 50 mL min^−1^ at a ramp rate of 10 °C min^−1^. DSC data (TA Instruments, Discovery 2500, New Castle, DE, USA) were collected from −60 to 200 °C at a ramp rate of 10 °C min^−1^. XRD patterns were determined using a Bruker diffractometer to elucidate the crystal structure of composite films with scanning from 10 to 80 2Θ. Chemical bonding environments of the films were determined using FTIR (Thermo Fisher, Waltham, MA, USA) and were recorded from 400 to 4000 cm^−1^.

### 4.3. Electrochemical Characterization

Prepared films were removed and punched into circular disks. The films were compressed between two stainless steel blocking electrodes in a compression cell that enables tunable pressure. The cell body was made out of PEEK and was purchased from MSE, then modified to include a fixture with springs to control pressure. Springs were 1.5 in initial length with a spring constant of 13.04 lbf/in, which was checked using Fujifilm pressure paper. Potentiostatic electrochemical impedance spectroscopy measurements were performed with a 10 mV applied AC voltage over a frequency range of 1 MHz to 100 Hz using a 1255 Solartron Analytical (Solartron Analytical, Oak Ridge, TN, USA) potentiostat. Temperature-dependent data were collected from 10 to 90 °C in an oven, with at least 30 min allowed for temperature to equilibrate. Ionic conductivity, σ (S cm^–1^), was determined from Equation (1), where L is the thickness of the compressed film, R is the charge transfer resistance from EIS, and S is the area of the film (1.09 cm^2^).

(1) $$\sigma =\frac{L}{R*S}$$

Energy of activation, E_a_, was calculated via the Arrhenius equation (Equation (3)), where R is the gas constant and T is the temperature.

(2) $$\sigma =\sigma_{0}\exp\left(\frac{-E_{a}}{\mathrm{RT}}\right)$$

Symmetric cells were prepared with Li electrodes (scraped to remove surface impurities) in 2032-coin cell format. Cells were cycled at a current density of 0.1 mA cm^−2^ at 50 °C for 1 h per charge/discharge. Li transference, t_Li+_, was determined at room temperature and calculated from the Bruce–Vincent–Evans Equation (Equation (2)) where I is the current at steady state (I_SS_) and initial (I_0_) conditions, R is the interfacial resistance at steady state (R_SS_) and initial (R_0_) conditions, and ΔV is the polarization potential applied. The time of applied polarization potential was chosen in order to reach steady state conditions (very little change in current decay profile).

(3) $$t_{+}=\frac{I_{\mathrm{ss}}(\Delta V-I_{0}R_{0})}{I_{0}(\Delta V-I_{\mathrm{ss}}R_{\mathrm{ss}})}$$

## Figures and Tables

**Table 1:** Physical properties and thermal properties of GPEs, determined from DSC thermograms.

Sample	Thickness (µm)	Solvent Retention (%, Calculated)	Solvent Retention (%, TGA)	Enthalpy (J g^−1^)	X_c_ (%)	T_melt,PVDF-HFP_ (°C)	T_melt,SN_ (°C)
PVDF-HFP	265	14.8	20.4	25.034	24.071	148.55	---
PVDF-HFP-SN	357	26.4	28.1	13.302	12.790	139.32	53.59
PVDF-HFP-SN-LLZTO	275	25.9	27.4	18.445	17.736	141.16	53.04

**Table 2:** Peaks from FTIR of GPES and approximate wavenumber.

Wavenumber (cm^−1^)	Peak Designation
709	PVDF-HFP, γ-phase
739	LiTFSI
812	PVDF-HFP, γ-phase
837	PVDF-HFP, β-phase
854	PVDF-HFP, α-phase
1056	PVDF-HFP, C–C
1173	PVDF-HFP, –CF_2_–
1353	LiTFSI
1407	PVDF-HFP, –CH_2_–

**Table 3:** Electrochemical properties of GPEs.

Sample	Ionic Conductivity at 25 °C (S cm^−1^ × 10^−5^)	Maximum Conductivity with Corresponding Pressure at 90 °C (S cm^−1^ × 10^−3^, psi)	E_a_ (eV)	t_Li+_
PVDF-HFP	0.16	0.47, 16.3	0.66	0.18
PVDF-HFP-SN	2.96	1.09, 6.43	0.46	0.95
PVDF-HFP-SN-LLZTO	4.14	1.32, 16.3	0.45	0.28

**Table 4:** Conductivities of GPEs prepared with PVDF-HFP and LLZO, where N/D refers to undetermined value.

LLZO (wt.%)	Conductivity (S cm^−1^)	Temperature (°C)	Solvent, Retention (%)	Test Cell	Reference
LLZTO, 5	1.3 × 10^−3^	90	NMP, 26	Pressure Cell	This Work
LLZTO, 5	4.1 × 10^−5^	Room Temperature	NMP, 26	Pressure Cell	This Work
LLZTO, 20	8.2 × 10^−4^ 4.9 × 10^−5^	60 20	NMP, N/D	CR3032 Coin Cell	[29]
LLZTO, 30	3.6 × 10^−4^	Room Temperature	NMP/Acetone, N/D	CR2032 Coin Cell	[24]
LLZTO, 30	7.9 × 10^−4^	Room Temperature	DMF, N/D	CR2025 Coin Cell	[30]
Ga-LLZO, 10	9.8 × 10^−5^	Room Temperature	DMF, N/D	“button cell”	[31]
Al-LLZTO, 20	5.4 × 10^−4^	Room Temperature	NMP, N/D	CR2025 Coin Cell	[32]

## Data Availability

The original contributions presented in this study are included in the article/Supplementary Materials. Further inquiries can be directed to the corresponding author.

## Supplementary Materials

The following supporting information can be downloaded at: https://www.mdpi.com/article/10.3390/gels11110890/s1, Figure S1: Temperature dependence of wet (non-vacuum dried) GPE films in a T-cell, Figure S2: Dependence of compressed film thickness and film compression on applied pressure, Figure S3: Nyquist plots of GPEs at variable temperature (10–90 °C) and optimal pressure, Figure S4: Resistance of GPEs at increasing temperature, then after cooling to room temperature. Figure S5: Resistance of GPEs at increasing temperature, then after cooling to room temperature (lighter colored points).

## References

[1] Zhang Z., Wang X., Li X., Zhao J., Liu G., Yu W., Dong X., Wang J. (2023). Review on composite solid electrolytes for solid-state lithium-ion batteries. Mater. Today Sustain..

[2] Zheng F., Kotobuki M., Song S., Lai M.O., Lu L. (2018). Review on solid electrolytes for all-solid-state lithium-ion batteries. J. Power Sources.

[3] Chaturvedi P., Choi D. (2025). Ambient-atmosphere processed flexible all-solid-state lithium-ion battery using flexible and robust hybrid solid electrolyte membrane. J. Alloys Compd..

[4] Chen F., Jing M.X., Yang H., Yuan W.Y., Liu M.Q., Ji Y.S., Hussain S., Shen X.Q. (2021). Improved ionic conductivity and Li dendrite suppression of PVDF-based solid electrolyte membrane by LLZO incorporation and mechanical reinforcement. Ionics.

[5] Zhang D., Liu Y., Yang S., Zhu J., Hong H., Li S., Xiong Q., Huang Z., Wang S., Liu J. (2024). Inhibiting Residual Solvent Induced Side Reactions in Vinylidene Fluoride-Based Polymer Electrolytes Enables Ultra-Stable Solid-State Lithium Metal Batteries. Adv. Mater..

[6] Halder B., Mohamed M.G., Kuo S.-W., Elumalai P. (2024). Review on composite polymer electrolyte using PVDF-HFP for solid-state lithium-ion battery. Mater. Today Chem..

[7] Hernández G., Lee T.K., Erdélyi M., Brandell D., Mindemark J. (2023). Do non-coordinating polymers function as host materials for solid polymer electrolytes? The case of PVdF-HFP. J. Mater. Chem. A.

[8] Cao S., Chen F., Shen Q., Zhang L. (2023). Dual-coordination-induced poly (vinylidene fluoride)/Li_6.4_Ga_0.2_La_3_Zr_2_O_12_/succinonitrile composite solid electrolytes toward enhanced rate performance in all-solid-state lithium batteries. ACS Appl. Mater. Interfaces.

[9] Wei T., Zhang Z.-H., Wang Z.-M., Zhang Q., Ye Y.-S., Lu J.-H., Rahman Z.U., Zhang Z.-W. (2020). Ultrathin solid composite electrolyte based on Li_6.4_La_3_Zr_1.4_Ta_0.6_O_12_/PVDF-HFP/LiTFSI/succinonitrile for high-performance solid-state lithium metal batteries. ACS Appl. Energy Mater..

[10] Qiu G., Shi Y., Huang B. (2022). A highly ionic conductive succinonitrile-based composite solid electrolyte for lithium metal batteries. Nano Res..

[11] Liu Q., Yang G., Li X., Zhang S., Chen R., Wang X., Gao Y., Wang Z., Chen L. (2022). Polymer electrolytes based on interactions between [solvent-Li+] complex and solvent-modified polymer. Energy Storage Mater..

[12] Zhang X., Liu T., Zhang S., Huang X., Xu B., Lin Y., Xu B., Li L., Nan C.-W., Shen Y. (2017). Synergistic Coupling between Li_6.75_La_3_Zr_1.75_Ta_0.25_O_12_ and Poly(vinylidene fluoride) Induces High Ionic Conductivity, Mechanical Strength, and Thermal Stability of Solid Composite Electrolytes. J. Am. Chem. Soc..

[13] Alam T., Mondal A., Das A. (2024). Role of LLZO dispersion in ion migration property of a ceramic integrated polymer composite electrolyte. Ionics.

[14] Zeng Y., Zhao L., Zhang J., Li Q., Sun D., Ren Y., Tang Y., Jin G., Wang H. (2023). La_2_O_3_ Filler’s Stabilization of Residual Solvent in Polymer Electrolyte for Advanced Solid-State Lithium-Metal Batteries. Small Sci..

[15] He W., Ding H., Chen X., Yang W. (2023). Three-dimensional LLZO/PVDF-HFP fiber network-enhanced ultrathin composite solid electrolyte membrane for dendrite-free solid-state lithium metal batteries. J. Membr. Sci..

[16] Prabakaran P., Manimuthu R.P., Gurusamy S., Sebasthiyan E. (2017). Plasticized polymer electrolyte membranes based on PEO/PVdF-HFP for use as an effective electrolyte in lithium-ion batteries. Chin. J. Polym. Sci..

[17] Molla S., Khatun F., Rajak U., Bagchi B., Das S., Thakur P. (2022). Electroactive CTAB/PVDF composite film based photo-rechargeable hybrid power cell for clean energy generation and storage. Sci. Rep..

[18] Tafur J.P., Santos F., Fernández Romero A.J. (2015). Influence of the ionic liquid type on the gel polymer electrolytes properties. Membranes.

[19] Tong R.-A., Chen L., Fan B., Shao G., Liu R., Wang C.-A. (2021). Solvent-Free Process for Blended PVDF-HFP/PEO and LLZTO Composite Solid Electrolytes with Enhanced Mechanical and Electrochemical Properties for Lithium Metal Batteries. ACS Appl. Energy Mater..

[20] Cai X., Lei T., Sun D., Lin L. (2017). A critical analysis of the α, β and γ phases in poly (vinylidene fluoride) using FTIR. RSC Adv..

[21] Arsyad A., Saaid F., Najihah M., Tan W. (2023). FTIR studies on interactions among components in PVdF-HFP: PC: MPII electrolytes. IOP Conference Series: Earth and Environmental Science.

[22] Lu J., Liu Y., Yao P., Ding Z., Tang Q., Wu J., Ye Z., Huang K., Liu X. (2019). Hybridizing poly (vinylidene fluoride-co-hexafluoropropylene) with Li_6.5_La_3_Zr_1.5_Ta_0.5_O_12_ as a lithium-ion electrolyte for solid state lithium metal batteries. Chem. Eng. J..

[23] Singh V.K., Singh R.K. (2015). Development of ion conducting polymer gel electrolyte membranes based on polymer PVdF-HFP, BMIMTFSI ionic liquid and the Li-salt with improved electrical, thermal and structural properties. J. Mater. Chem. C.

[24] Han B., Jiang P., Li S., Lu X. (2021). Functionalized gel polymer electrolyte membrane for high performance Li metal batteries. Solid State Ion..

[25] Ruan Z., Du Y., Pan H., Zhang R., Zhang F., Tang H., Zhang H. (2022). Incorporation of poly (ionic liquid) with PVDF-HFP-based polymer electrolyte for all-solid-state lithium-ion batteries. Polymers.

[26] Duan T., Cheng H., Liu Y., Sun Q., Nie W., Lu X., Dong P., Song M.-K. (2024). A multifunctional Janus layer for LLZTO/PEO composite electrolyte with enhanced interfacial stability in solid-state lithium metal batteries. Energy Storage Mater..

[27] Liu Z., Zhang S., Zhou Q., Zhang Y., Lv D., Shen Y., Fu X., Wang X., Luo S., Zheng Y. (2023). Insights into quasi solid-state polymer electrolyte: The influence of succinonitrile on polyvinylene carbonate electrolyte in view of electrochemical applications. Battery Energy.

[28] Voigt N., van Wüllen L. (2014). The effect of plastic-crystalline succinonitrile on the electrolyte system PEO: LiBF4: Insights from solid state NMR. Solid State Ion..

[29] Yadav P., Hosen M.S., Dammala P.K., Ivanchenko P., Van Mierlo J., Berecibar M. (2023). Development of composite solid polymer electrolyte for solid-state lithium battery: Incorporating LLZTO in PVDF-HFP/LiTFSI. Solid State Ion..

[30] Wu X., Jie X., Liang X., Zhang L., Wang J., Wu S. (2024). Polymer/ceramic gel electrolyte with in-situ interface forming enhances the performance of lithium metal batteries. J. Energy Storage.

[31] Cai J., Liu T., Liu C., Liu G. (2023). PVDF-HFP/LiTFSI based composite solid state electrolyte with different micromorphology of Li_6.25_Ga_0.25_La_3_Zr_2_O_12_ doping. J. Alloys Compd..

[32] Zou J., Gao X., Zhou X., Yang J., Tang J., Kou H., Chang R., Zhang Y. (2023). Al and Ta co-doped LLZO as active filler with enhanced Li+ conductivity for PVDF-HFP composite solid-state electrolyte. Nanotechnology.

[33] Hu J., He P., Zhang B., Wang B., Fan L.-Z. (2020). Porous film host-derived 3D composite polymer electrolyte for high-voltage solid state lithium batteries. Energy Storage Mater..

[34] Zha W., Li J., Li W., Sun C., Wen Z. (2021). Anchoring succinonitrile by solvent-Li+ associations for high-performance solid-state lithium battery. Chem. Eng. J..

[35] Noh H., Kim D., Lee W., Jang B., Ha J.S., Yu J.H. (2023). Surface Modification of Ga-Doped-LLZO (Li_7_La_3_Zr_2_O_12_) by the Addition of Polyacrylonitrile for the Electrochemical Stability of Composite Solid Electrolytes. Energies.

[36] Counihan M.J., Lee J., Mirmira P., Barai P., Burns M.E., Amanchukwu C.V., Srinivasan V., Zhang Y., Tepavcevic S. (2025). Improved interfacial Li-ion transport in composite polymer electrolytes via surface modification of LLZO. Energy Mater..

[37] Diederichsen K.M., McShane E.J., McCloskey B.D. (2017). Promising routes to a high Li+ transference number electrolyte for lithium ion batteries. ACS Energy Lett..

[38] Zhang Q., Liu K., Liu K., Li J., Ma C., Zhou L., Du Y. (2020). Study of a composite solid electrolyte made from a new pyrrolidone-containing polymer and LLZTO. J. Colloid Interface Sci..

[39] Maeyoshi Y., Yoshii K., Sano H., Sakaebe H., Tamate R., Kaneko T., Sodeyama K. (2025). Gel Polymer Electrolytes Based on Poly (vinylidene fluoride-co-hexafluoropropylene) and Salt-Concentrated Electrolytes for High-Voltage Lithium Metal Batteries. ACS Appl. Polym. Mater..

