# Supplementary material for: The Effects of Processing Conditions and Pressure on Composite Polymer Electrolyte Performance
Source: Gels. 2025 Nov 5;11(11):890. doi: 10.3390/gels11110890 (PMC12652269; doi:10.3390/gels11110890)
Supplement: Supplementary file 1 [file gels-11-00890-s001.zip › gels-3910835-supplementary.pdf]

Supporting Information

Elucidating the Effects of Processing and Testing Conditions on Composite Polymer Electrolyte Properties

Samantha Macchi, Lillian N. Elam, Josefine D. McBrayer, Noah B. Schorr*

Power Sources R&D, Sandia National Laboratories, Albuquerque, NM 87185 USA

**Corresponding author*

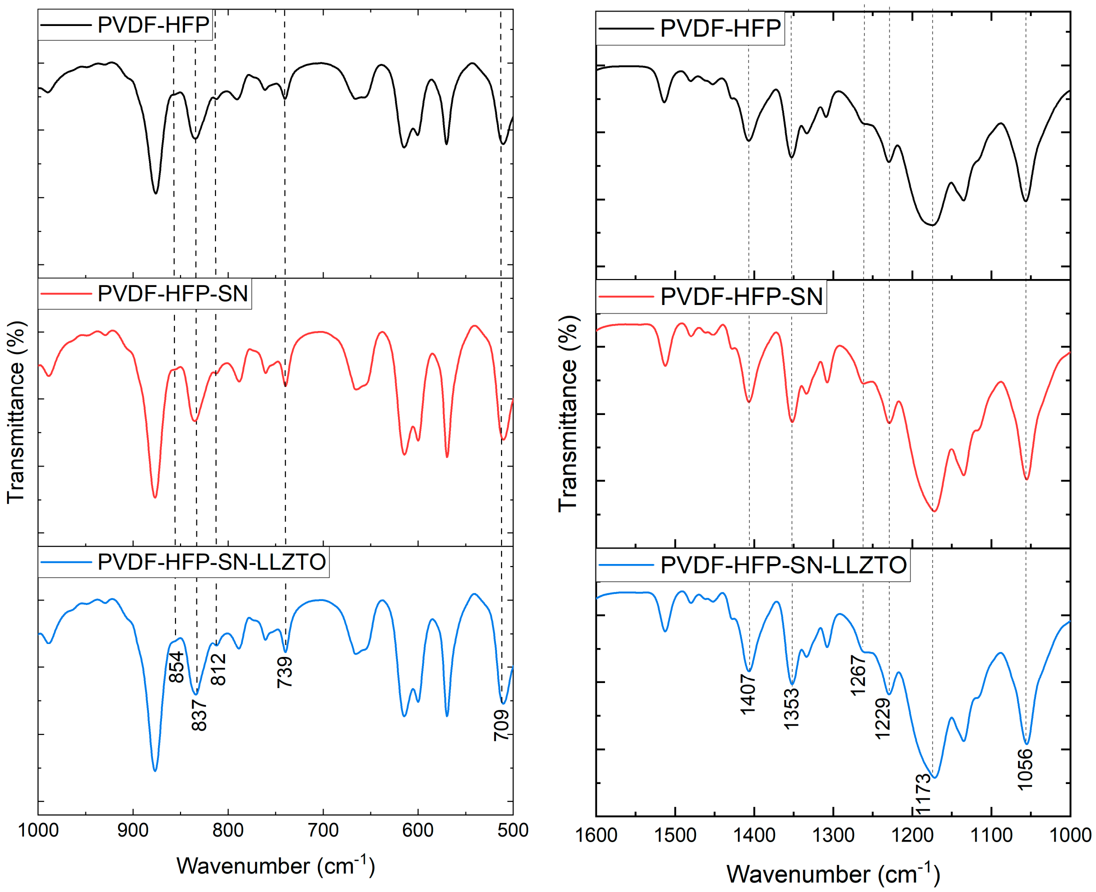

Figure S1. FTIR spectra of GPEs showing primary peak locations.

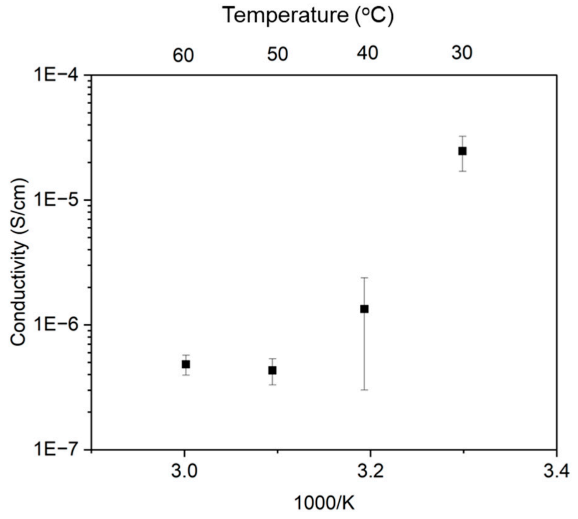

Figure S2. Temperature dependence of wet (non-vacuum dried) GPE films in a T-cell.

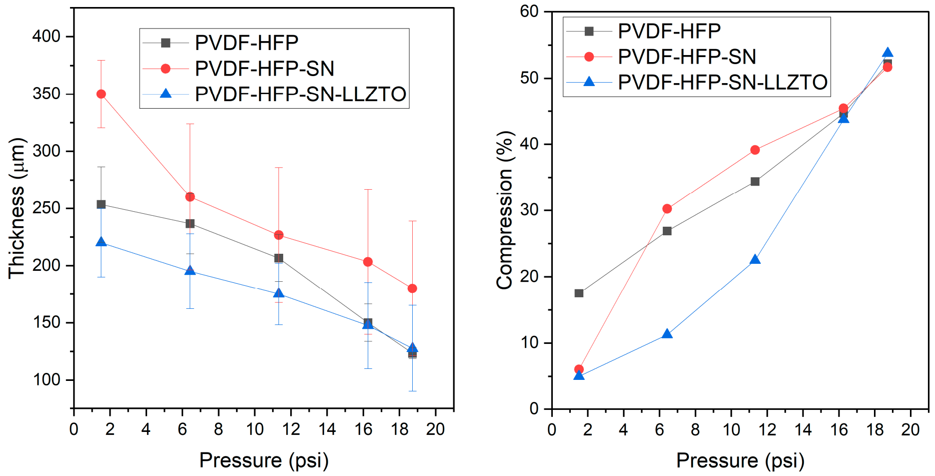

Figure S3. Dependence of compressed film thickness (left) and film compression (right) on applied pressure.

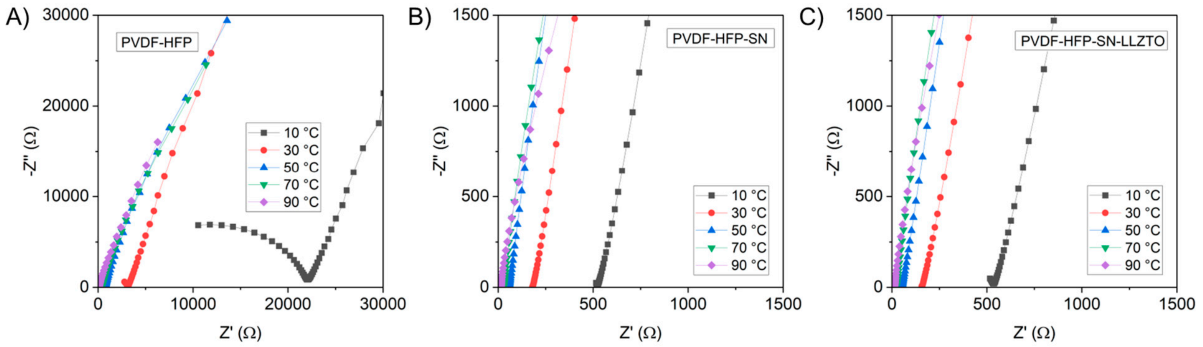

Figure S4. Nyquist plots of GPEs at variable temperature (10-90 °C) and optimal pressure (16.3 psi for PVDF-HFP and PVDF-HFP-SN-LLZTO and 6.43 psi for PVSD-HFP-SN).

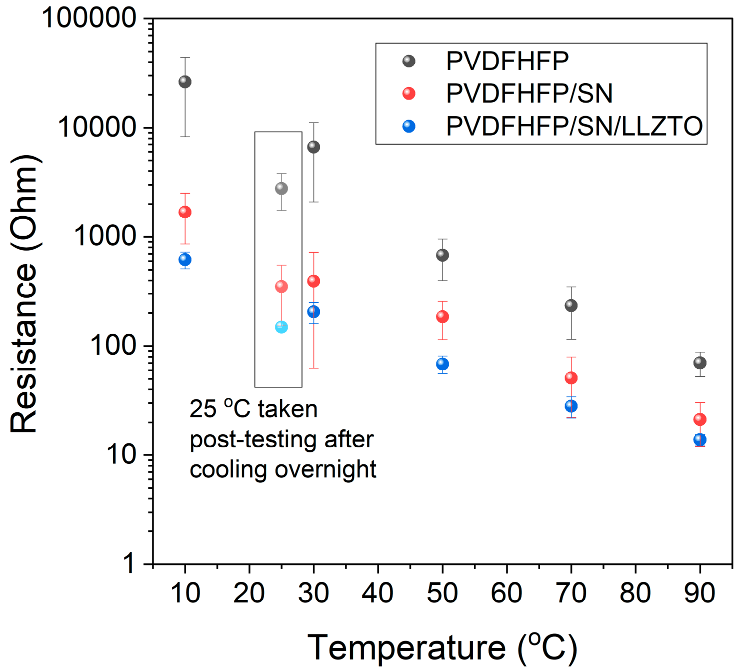

Figure S5. Resistance of GPEs at increasing temperature, then after cooling to room temperature (lighter colored points).
